# Characterization of Calcium-Dependent Protein Kinases 3, a Protein Involved in Growth of *Cryptosporidium parvum*

**DOI:** 10.3389/fmicb.2020.00907

**Published:** 2020-05-08

**Authors:** Qiang Zhang, Yaqiong Guo, Na Li, Yu Li, Jiayuan Su, Rui Xu, Ziding Zhang, Yaoyu Feng, Lihua Xiao

**Affiliations:** ^1^State Key Laboratory of Bioreactor Engineering, School of Resource and Environmental, East China University of Science and Technology, Shanghai, China; ^2^Key Laboratory of Zoonosis of Ministry of Agriculture, College of Veterinary Medicine, South China Agricultural University, Guangzhou, China; ^3^State Key Laboratory of Agrobiotechnology, College of Biological Sciences, China Agricultural University, Beijing, China

**Keywords:** *Cryptosporidium parvum*, calcium-dependent protein kinase 3, inhibitor, enzyme, growth

## Abstract

Calcium-dependent protein kinases (CDPKs) are considered promising targets for pharmaceutical intervention of cryptosporidiosis. Whole-genome sequencing has revealed the presence of several CDPKs (*Cp*CDPKs) in *Cryptosporidium parvum*. In this study, we expressed recombinant *Cp*CDPK3 encoded by the *cgd5_820* gene in *Escherichia coli*. The biologic characteristics and functions of *Cp*CDPK3 were examined using qRT-PCR, immunofluorescence microscopy, and *in vitro* neutralization assay. The expression of the *cgd5_820* gene peaked in merozoites during *in vitro* culture while the *Cp*CDPK3 protein was expressed in both sporozoites and merozoites. Polyclonal antibodies against *Cp*CDPK3 showed no significant inhibitory effects on host invasion by the parasites. We assessed the inhibitory effects of 46 candidate compounds from molecular docking of *Cp*CDPK3 on both *C. parvum* development and *Cp*CDPK3 enzyme activities. One compound was identified to be effective. Results of these analyses suggest that *Cp*CDPK3 might play an important role in the growth of *C. parvum*.

## Introduction

*Cryptosporidium* spp. are apicomplexan parasites, causing moderate-to-severe diarrhea in both humans and animals worldwide (Kotloff et al., 2013; Checkley et al., 2015). *Cryptosporidium* infection is self-limiting in immunocompetent hosts but can have prolonged and detrimental effects on immunocompromised hosts such as HIV/AIDS patients and transplant recipients (Wang et al., 2018; Bones et al., 2019). Currently, nitazoxanide is the only drug approved by the U.S. Food and Drug Administration against cryptosporidiosis, although it is ineffective in malnourished children and AIDS patients (Abubakar et al., 2007, 2010; Amadi et al., 2009). The lack of effective treatment is partially attributed to our limited knowledge of the invasion and intracellular development of *Cryptosporidium* spp. (Bhalchandra et al., 2018).

Calcium is involved in several critical events in the life cycle of apicomplexan parasites, including protein secretion, gliding motility, cell invasion, and egress (Billker et al., 2009). In these pathogens, calcium-dependent protein kinases (CDPKs) are the most abundant class of calcium sensors, being found in apicomplexan protozoa, ciliates, and plants, but not in fungi and vertebrates (Harper and Alice, 2005). As a result, they are considered attractive drug targets for cryptosporidiosis (Hui et al., 2015). Thus far, whole-genome sequencing and RNA-Seq analysis have identified 11 CDPKs in *Cryptosporidium parvum* (Lippuner et al., 2018).

Most previous studies of CDPKs of *C. parvum* (*Cp*CDPKs) are on *Cp*CDPK1, which is expressed in all life cycle stages, and believed to play an important role in the invasion (Castellanos-Gonzalez et al., 2016; Kuhlenschmidt et al., 2016) and possibly growth of *C. parvum* (Huang et al., 2017). In comparison, the function of *Cp*CDPK3, which shares high structural homology with *Cp*CDPK1, is still poorly understood. In other apicomplexans, *Toxoplasma gondii* CDPK3 (*Tg*CDPK3) was shown to play an essential role in the egress of the pathogen out of host cells (Lourido et al., 2012; Mccoy et al., 2012), while *Plasmodium falciparum* CDPK1 (*Pf*CDPK1, homologous to *Cp*CDPK3) was demonstrated to participate in the egress of merozoites from schizonts (Kato et al., 2008).

To explore the functions of *Cp*CDPK3, we have expressed in the study the recombinant protein of *Cp*CDPK3 encoded by the *cgd5_820* gene, and examined its potential role in the life cycle of *C. parvum*. In addition, one potential inhibitor of the enzyme was identified in the process.

## Materials and Methods

### Parasite and Cell Culture

*Cryptosporidium parvum* oocysts (IOWA isolate) were purchased from Waterborne, Inc. (New Orleans, LA, United States) and stored in phosphate-buffered saline (PBS) with antibiotics at 4°C. All oocysts used in this study were stored for less than 3 months. Before usage, oocysts were treated on ice with chilled 0.5% sodium hypochlorite for 10 min and washed three times afterward with PBS by centrifugation at 13,200 × *g* for 2 min.

Human colon adenocarcinoma cells (HCT-8 cells) were purchased from the cell bank of the Chinese Academy of Sciences. They were cultured in RPMI 1640 medium supplemented with 10% fetal bovine serum (FBS), 100 U/mL penicillin, and 100 μg/mL streptomycin at 37°C and 5% CO_2_.

### Cloning, Expression, and Purification of Recombinant *Cp*CDPK3 and Preparation of Polyclonal Antibodies

The full-length *cgd5_820* gene (Gene ID: 3373302) was amplified using PCR from genomic DNA of the *C. parvum* IOWA isolate. The primers used included CDPK3-F1 5′-CGCGGATCCATATCACTTTTTATTCAAAAG-3′ (with *Bam*H I restriction enzyme site underlined) and CDPK3-R1 5′-CCGCTCGAGATTTTTTTTGAGCTGGGGTT-3′ (with *Xho* I restriction enzyme site underlined). The PCR product was purified using the E.Z.N.A.^®^ Cycle-Pure Kit (Omega Bio-Tek, Norcross, GA, United States), digested with restriction enzymes *Bam*H I and *Xho* I (New England Biolabs, Ipswich, MA, United States), and ligated into the pET-28a-c(+) vector (Novagen, Madison, WI, United States). The ligation product was used to transform the DH5α competent cells of *Escherichia coli*. The positive colonies were identified using PCR and DNA sequence analyses. The recombinant vectors were extracted from the DH5α cells using E.Z.N.A.^®^ Plasmid Mini Kit (Omega Bio-Tek).

For the expression of recombinant restriction enzymes, *E. coli* BL21(DE3) competent cells were transformed with the recombinant *Cp*CDPK3-pET-28a-c(+) vector and cultured in LB medium supplemented with 100 μg/mL kanamycin. The expression of *Cp*CDPK3 was induced by adding 0.5 mM isopropylthio-β-galactoside (IPTG) to the culture, which was maintained at 25°C for 8 h. The expression level was examined using SDS-PAGE and Western blot analyses with anti-His-tag antibodies.

For protein purification, the BL21(DE3) cultures were collected by centrifugation and lyzed by sonication on ice. The lysate was centrifuged, and the supernatant generated was filtered through a 0.45-μm polyvinylidene fluoride (PVDF) membrane filter (Millipore, Billerica, MA, United States). The filtrate was loaded onto a column containing Ni-NTA His-bind resins (Novagen) at room temperature. *Cp*CDPK3 was eluted from the resins with 100 mM imidazole buffer and examined using SDS-PAGE and Western blot analyses. The matrix-assisted laser desorption/ionization time of flight mass spectrometry (MALDI-TOF/MS) was used to analyze the SDS-PAGE bands at the Applied Protein Technology Corporation (Shanghai, China) for the verification of the identity of the expressed protein.

Polyclonal antibodies against recombinant *Cp*CDPK3 were generated through immunizations of specific pathogen-free rabbits using Freund’s complete and incomplete adjuvants by the GenScript Corporation (Nanjing, China). After the final immunization, the serum of rabbits was collected, and the *Cp*CDPK3-specific antibodies were purified by affinity chromatography with purified recombinant protein. The titer and specificity of the antibodies were assessed using enzyme-linked immunosorbent assay (ELISA) and Western blot analysis, respectively.

### Assessment of *cgd5_820* Expression in Developmental Stages

The expression of the *cgd5_820* gene in intracellular stages of *C. parvum* was assessed using qRT-PCR as described (Mauzy et al., 2012). HCT-8 cells were cultured in 12-well plates until 60% confluence. Prior to infection, the culture medium was replaced by RPMI 1640 containing 2% FBS. Sodium hypochlorite-treated oocysts were inoculated onto cells (5 × 10^5^ oocysts/well) and incubated at 37°C for 2 h. The unexcysted and free sporozoites were washed off the cells with PBS. The cells were further cultured in fresh medium with 2% FBS. Total RNA was isolated from cells at 2, 6, 12, 24, 36, 48, and 72 h post-infection using the RNeasy Mini kit (QIAGEN, Hilden, Germany), and reverse-transcribed by using the RevertAid First Strand cDNA Synthesis Kit (Thermo Fisher Scientific, Waltham, MA, United States). The qPCR was conducted in 20-μL reaction mixture which contained 1 μL cDNA, 0.5 mM primers, and 10 μL 2 × SYBR Green Real-Time PCR Master Mix (Toyobo, Osaka, Japan) in a Light Cycler 480 Instrument II (Roche, Basel, Switzerland). The c*gd5_820* gene was amplified by using the primers CDPK3-F2 (5′-CGAATGGAAGAATGTCTCTGAA-3′) and CDPK3-R2 (5′-AGGCTTGGTAGCTCAATACCTG-3′). Data from the *C. parvum* 18S rRNA gene were used in data normalization as described (Mauzy et al., 2012). Each cDNA was analyzed by qPCR in duplicate. The relative expression level of the *cgd5_820* gene at different time points was calculated with the 2^–ΔΔ*C*_T_^ method (Livak and Schmittgen, 2001). The results were based on the mean values from three independent biological experiments.

### Assessment of Expression of Native *Cp*CDPK3

For excystation, oocysts were treated with 0.5% sodium hypochlorite as described above and incubated with D-Hanks buffer containing 0.25% trypsin and 0.75% sodium taurocholate at 37°C for 1 h. The released sporozoites were collected and washed by centrifugation at 13,200 × *g* for 2 min. They were resuspended in PBS, mixed with protease inhibitor cocktail (Merck, Darmstadt, Germany) and 5× protein loading buffer, and incubated in a 100°C water bath for 5 min. The native proteins in the lysate were separated by SDS-PAGE (∼5 × 10^6^ oocysts/lane), transferred onto PVDF membranes, and probed with anti-*Cp*CDPK3 antibodies (0.45 μg/mL), antiserum (1:4000 dilution), or pre-immune serum (1:4000 dilution). Horse radish peroxidase-conjugated anti-rabbit IgG (Cell Signaling Technology, Beverly, MA, United States) was used as the secondary antibody. The reactivity was visualized using an ECL system (Tanon, Shanghai, China).

### Assessment of *Cp*CDPK3 Expression in Developmental Stages

For the assessment of *Cp*CDPK3 expression using immunofluorescence microscopy, *C. parvum* oocysts and excysted sporozoites were fixed with methanol for 20 min on SuperStick Slides (Waterborne). For the collection of intracellular stages, HCT-8 cells cultured on coverslips were infected with *C. parvum* as described above and maintained for 24 and 48 h. After fixation with methanol, oocysts, sporozoites, and cultured cells were permeabilized with 0.5% Triton X-100 in PBS for 15 min, blocked with 5% bovine serum albumin (BSA) in PBS for 1 h, and incubated with anti-*Cp*CDPK3 antibodies (0.45 μg/mL) for 1 h. Alexa Fluor*textregistered* 594-conjugated Goat Anti-rabbit IgG (Cell Signaling Technology) was used as the secondary antibody at 1:400. After incubation for 1 h, the cell nuclei were counterstained with the 4,6-diamidino-2-phenylindole (DAPI). Three PBS washes were performed after each treatment of the slides or coverslips. The slides and coverslips were examined under an Olympus BX53 fluorescence microscope (Olympus, Tokyo, Japan).

### *In vitro* Neutralization of *C. parvum* Invasion With Anti-*Cp*CDPK3 Antibody

*In vitro* neutralization assay was used to assess the involvement of *Cp*CDPK3 in *C. parvum* invasion. Briefly, excysted sporozoites were incubated at 37°C in medium containing 1:200, 1:500, and 1:1,000 dilutions of post-immune serum or pre-immune serum for 15 min. They were added onto HCT-8 cells cultured on coverslips at 1 × 10^5^ oocysts/coverslip. After 2-h incubation, the culture was washed with PBS three times and allowed to continue for 24 h. The developmental stages of *C. parvum* in cells were stained with Cy3-labeled Sporo-Glo^TM^ antibody (Waterborne) and examined under a BX53 fluorescence microscope. For each slide, images of 50 random fields were captured under 200×. The number of parasites in each field was quantified using the Image J software^1^. The mean value was used to calculate the parasite load. Data from cells treated with pre-immune serum in corresponding dilutions were used as control. All experiments were performed in triplicate.

### Inhibition of *C. parvum* Invasion and Growth With Candidate Inhibitors of *Cp*CDPK3

A total of 50 small molecules were selected from the ChemDiv database through the molecular docking of the *Cp*CDPK3 structure (Table 1). The binding abilities of the small molecules to *Cp*CDPK3 were scored according to the binding energy, which was based on ligand efficiency, Coulomb energy, Van der waals energy, and H-bond energy. The *C. parvum* culture system was used to assess the anti-cryptosporidial effect of 46 commercially available small molecules, using qRT-PCR in the quantitation of parasites (Zhang et al., 2012). Briefly, HCT-8 cells were cultured in 96-well plates until 80% confluence. Excysted sporozoites were incubated with 10 μM compounds or DMSO in RPMI 1640 culture medium with 2% FBS for 15 min and added to the cell monolayer at 1 × 10^5^ oocysts/well. After 2-h incubation, free sporozoites were washed off with PBS. The cultures were allowed to continue in medium containing compounds or DMSO for 24 h. Total RNA was extracted from the cultures using the RNeasy Mini kit (QIAGEN, Hilden, Germany). A HiScript II One Step qRT-PCR SYBR Green Kit (Vazyme, Nanjing, China) was used to quantitate the parasite load as described (Cai et al., 2005). At least two technical replicates were used in qRT-PCR analysis of each culture. In secondary analysis of selective compounds, various concentrations (from 20 nM to 25 μM) of the compounds were used to treat the *C. parvum* cell culture. All infection experiments in the study were performed in triplicate. To evaluate the cytotoxicity of the most effective compound, various concentrations (20 nM to 25 μM) of the compound were used to treat non-infected host cells cultured in 96-well plates for 24 h. The effect of the treatment on the cells was measured using a Cell Titer 96 AQueous One Solution Cell Proliferation Assay (MTS assay).

**TABLE 1:** Candidate compounds selected based on molecular docking of *Cp*CDPK3.

**Index**	**Name**	**Docking score**	**Ligand efficiency**	**Coulomb energy**	**Van der waals energy**	**H-bond energy**
1	3874-0007	−9.11	−0.38	−16.71	−39.62	−0.92
2	G856-0764	−8.95	−0.45	−14.39	−30.80	−0.85
3	F825-0963	−8.87	−0.32	−19.84	−32.65	−1.03
4	D074-0339	−8.87	−0.33	−15.51	−35.76	−0.99
5	D126-0056	−8.67	−0.31	−13.34	−35.58	−1.27
6	J106-0163	−8.66	−0.32	−10.61	−37.97	−0.58
7	D090-0041	−8.66	−0.54	−10.06	−24.25	−0.76
8	M510-0343	−8.59	−0.39	−13.86	−31.05	−0.61
9	D359-0714	−8.52	−0.27	−13.89	−37.89	−0.58
10	C276-1710	−8.45	−0.38	−17.71	−27.86	−0.67
11	D718-1386	−8.41	−0.40	−13.68	−29.74	−0.68
12	2516-3991	−8.41	−0.42	−15.21	−31.39	−0.62
13	8109-9134	−8.40	−0.44	−13.28	−28.19	−0.79
14	S636-0103	−8.36	−0.46	−14.23	−27.69	−0.60
15	P814-5429	−8.26	−0.34	−12.41	−33.34	−0.61
16	8019-7983	−8.26	−0.31	−16.96	−31.54	−0.72
17	D074-0541	−8.24	−0.32	−8.17	−35.86	−0.86
18	F742-0261	−8.23	−0.30	−13.58	−39.02	−0.61
19	Y041-6944	−8.22	−0.34	−14.98	−31.90	−0.61
20	C200-7971	−8.18	−0.36	−6.00	−37.12	−0.59
21	D074-0338	−8.14	−0.30	−14.99	−38.08	−0.98
22	D361-0070	−8.14	−0.37	−10.70	−26.35	−0.70
23	8020-2455	−8.13	−0.34	−14.85	−32.89	−0.54
24	Y040-6497	−8.13	−0.48	−11.22	−23.46	−0.81
25	D126-0065	−8.11	−0.27	−13.92	−37.76	−0.93
26	G821-3004	−8.11	−0.32	−12.57	−37.21	−0.67
27	S632-6222	−8.11	−0.31	−18.57	−27.60	−0.69
28	J106-0278	−8.10	−0.29	−14.08	−27.70	−0.75
29	G072-0343	−8.10	−0.45	−15.66	−28.19	−0.85
30	7999-4509	−8.07	−0.40	−16.95	−26.73	−1.02
31	D225-0067	−8.06	−0.31	−11.03	−34.60	−0.69
32	7999-4518	−8.05	−0.34	−10.98	−36.66	−0.80
33	M333-0125	−8.04	−0.33	−12.67	−36.08	−0.65
34	2516-4740	−8.02	−0.40	−15.20	−27.33	−0.74
35	R052-0872	−8.01	−0.42	−15.47	−23.75	−0.97
36	S576-0109	−8.00	−0.35	−14.38	−33.82	−0.86
37	2516-4740	−8.00	−0.40	−12.00	−28.69	−1.20
38	C066-5502	−7.99	−0.47	−11.44	−27.63	−0.30
39	P610-0073	−7.97	−0.47	−10.68	−26.69	−0.72
40	Y020-3483	−7.96	−0.36	−13.93	−33.06	−0.43
41	G856-0728	−7.94	−0.33	−10.98	−36.64	−0.69
42	F688-0008	−7.94	−0.36	−15.30	−30.58	−0.84
43	C797-1619	−7.94	−0.36	−15.41	−27.50	−0.27
44	8004-9286	−7.91	−0.29	−12.45	−27.64	−1.14
45	P174-0133	−7.88	−0.34	−12.33	−32.38	−0.61
46	M333-0438	−7.88	−0.32	−9.60	−36.79	−0.55
47	F083-0116	−7.86	−0.33	−5.41	−37.58	−0.61
48	P759-5054	−7.85	−0.27	−7.60	−35.88	−0.69
49	3042-5189	−7.84	−0.30	−16.46	−29.98	−0.61
50	5516-0721	−7.79	−0.27	−19.79	−26.98	−0.61

The anti-cryptosporidial effect of the active compound selected above was further evaluated using both *C. parvum* invasion and growth assays. In invasion experiments, a traditional *in vitro* neutralization assay based on immunofluorescence quantitation of parasite loads was used to evaluate the inhibition of *C. parvum* invasion with the method described in Section “*In vitro* Neutralization of *C. parvum* Invasion With Anti-CpCDPK3 Antibody.” In growth experiments, sporozoites were inoculated onto cell monolayer (1 × 10^5^ oocysts/well) and incubated for 2 h at 37°C. Afterward, free sporozoites were washed off with PBS. The cultures were allowed to continue in medium containing active compounds or DMSO for 48 h. The total RNA was extracted and used to evaluate the inhibition of *C. parvum* growth with the qRT-PCR method described above. All experiments were performed in triplicate.

### Assessment of Enzyme Activities of *Cp*CDPK3

The kinase activity of *Cp*CDPK3 was measured using an NADH-coupled ATPase assay with Syntide-2 (a peptide with the sequence PLARTLSVAGLPGKK) as a substrate as described previously (Dolle and Ziegler, 2009; Wernimont et al., 2010). For this, 400 μM Syntide-2, 400 μM ATP, 150 μM NADH, 300 μM phosphoenolpyruvic (PEP) acid, and a mixture of pyruvate kinase and lactate dehydrogenase (PK/LDH) from Sigma (with 4 units/mL PK and 6 units/mL of LDH) were incubated at 30°C for 15 min in reaction buffer containing 20 mM Tris, 30 mM NaCl, 10 mM MgCl_2_, 1 mM CaCl_2_, 10 mM DTT, 2 μg/mL BSA, and 0.01% Tween 20. The reaction was initiated by adding 75 nM *Cp*CDPK3 and measured at 340 nm with an Infinite*textregistered* 200 PRO multimode plate reader (Tecan, AG, Switzerland). A recombinant insulinase-like protease of *C. parvum*, INS-15, was used as the negative control in the kinase assay. For inhibitor screening and IC_50_ determination, 60 μM NADH, 300 μM PEP, PK/LDH (with 4 units/mL PK and 6 units/mL of LDH), 75 nM *Cp*CDPK3, and different concentrations (20 nM to 25 μM) of selective compounds were incubated in reaction buffer at 30°C for 15 min. DMSO was used as another control in this experiment. The reaction was initiated by adding a mixture of 50 μM Syntide-2 and 50 μM ATP. The data obtained were analyzed using the Student’s *t*-test and dose–response test (variable slope) implemented in GraphPad Prism^2^.

## Results

### Production of Recombinant *Cp*CDPK3 in *E. coli*

**FIGURE 1:**
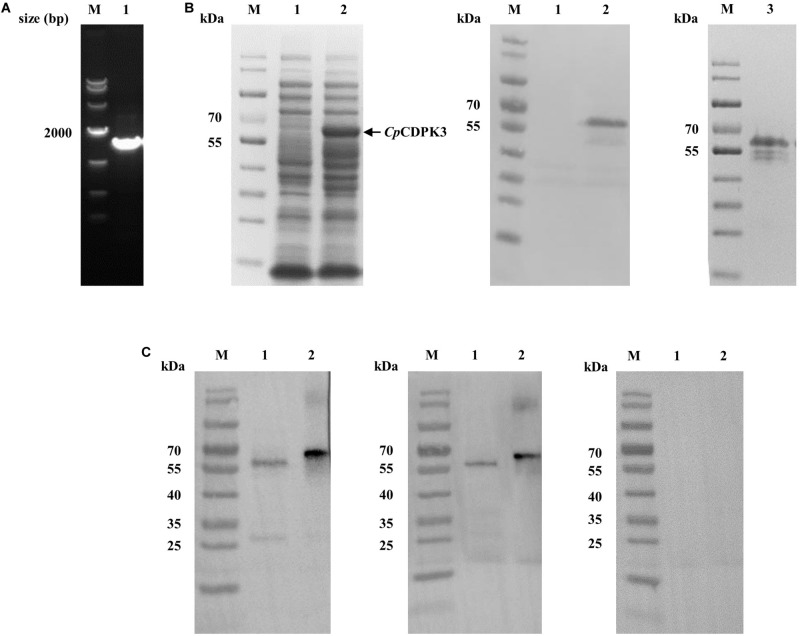
Production of recombinant *Cp*CDPK3 and polyclonal antibodies. **(A)** PCR amplification of the *cgd5_820* gene of *Cryptosporidium parvum*. Lane M: 1000 bp molecular markers; Lane 1: *cgd5_820* gene PCR product. **(B)** Expression and purification of recombinant *Cp*CDPK3. Recombinant *Cp*CDPK3 protein expressed in *E. coli* BL21 (DE3) was analyzed by SDS-PAGE (left panel) and Western blot (middle panel), while purified *Cp*CDPK3 was analyzed by SDS-PAGE alone (right panel). Lane M: protein molecular weight markers; Lane 1: lysate from culture of recombinant bacteria without IPTG induction; Lane 2: lysate from 8 h IPTG-induced culture of recombinant bacteria, with the expected product indicated by the black arrow; Lane 3: *Cp*CDPK3 purified by using Ni-NAT affinity chromatography. **(C)** Expression of native *Cp*CDPK3 protein in *C. parvum* sporozoites. The protein was identified by Western blots with post-immune serum (left panel), purified anti-*Cp*CDPK3 antibodies (middle panel), and pre-immune serum (right panel). Lane M: protein molecular weight markers; Lane 1: crude protein extracted from sporozoites. Lane 2: purified recombinant *Cp*CDPK3 protein.

The full-length *cgd5_820* gene encoding *Cp*CDPK3 was amplified from genomic DNA of *C. parvum* (Figure 1A) and cloned into the pET-28a-c(+) vector. The protein was expressed as the expected size of ∼66 kDa and confirmed using Western blot analysis with anti-His tag antibodies (Figure 1B) and MALDI-TOF/MS analysis. The latter obtained only peptide sequences of *Cp*CDPK3 (data not shown). The recombinant *Cp*CDPK3 protein was used in the generation of polyclonal antibodies and immune sera, which were used in further analysis of the purified recombinant *Cp*CDPK3 and native *Cp*CDPK3 in crude protein extract of sporozoites, with the pre-immune serum being used as the control. The purified recombinant *Cp*CDPK3 was recognized by the antibodies and immune serum at the expected size of ∼ 66 kDa, while the native *Cp*CDPK3 was recognized at a smaller size of ∼60 kDa. In the analysis of native proteins, a ∼30 kDa band was also recognized by polyclonal antibodies but not by the immune serum (Figure 1C).

### Expression of *Cp*CDPK3 in Developmental Stages

**FIGURE 2:**
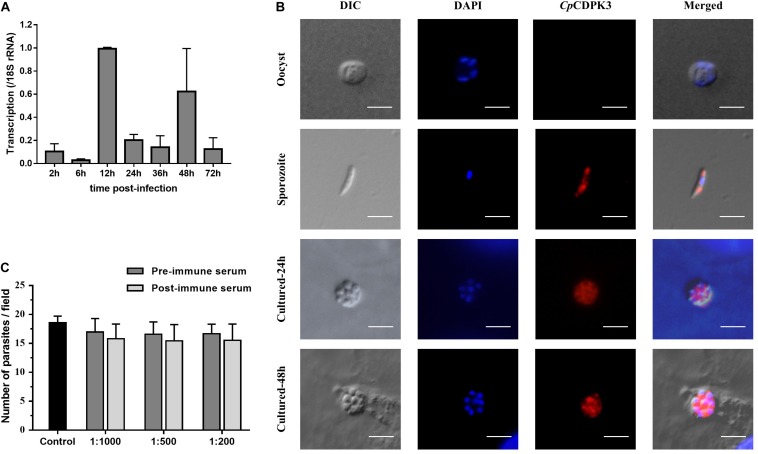
Expression and neutralization of *Cp*CDPK3 in sporozoites and developmental stages in *Cryptosporidium parvum*. **(A)** Relative expression levels of the *cgd5_820* gene at various time points of *C. parvum* culture. The gene expression was assessed using qPCR, with data from the *Cryptosporidium* 18S rRNA gene being used as an internal control for data normalization. Data presented are mean ± SD from three independent assays. **(B)** Expression of *Cp*CDPK3 in oocysts (first panel), sporozoites (second panel), and intracellular developmental stages of *C. parvum* in HCT-8 cell cultures at 24 h (third panel) and 48 h (fourth panel). The images were taken under differential interference contrast (DIC), with nuclei being counter-stained with 4’, 6-diamidino-2-phenylindole (DAPI), parasites stained by immunofluorescence with Alexa 594-labeled *Cp*CDPK3 (*Cp*CDPK3), and superimposition of the three images (Merged). Bars = 5 μm. **(C)** Neutralization efficiency of *C. parvum* invasion by post-immune serum against *Cp*CDPK3. Data from cultures treated with the pre-immune serum were used for data normalization of infection level. Data presented are mean ± SD from three independent assays.

The expression level of the *cgd5_820* gene in intracellular developmental stages of *C. parvum* was assessed using qRT-PCR analysis of RNA extracted from infected HCT-8 cells. After infection of the cells with sporozoites, low expression of the *cgd5_820* gene was observed at 2, 6, 24, 36, and 72 h (Figure 2A). The highest expression of the gene occurred at 12 and 48 h post-infection.

The expression of the *Cp*CDPK3 protein in life cycle stages was examined using immunofluorescence microscopy. The intact *C. parvum* oocysts did not react with the anti-*Cp*CDPK3 antibodies (Figure 2B, first panel). In contrast, the antibodies reacted with almost the entire excysted sporozoites (Figure 2B, second panel). At 24 and 48 h post-infection, the antibodies appeared to react with the entire merozoites (Figure 2B, third and fourth panels). The *Cp*CDPK3 antibodies did not recognize the parasitophorous vacuole at both time points (Figure 2B, third and fourth panels).

### Poor Neutralization of *C. parvum* Invasion by Anti-*Cp*CDPK3 Antibodies

An *in vitro* neutralization assay was used to assess the effect of anti-*Cp*CDPK3 antibodies on *C. parvum* invasion of HCT-8 cells. There was no significant reduction in parasite load in cultures treated with the immune serum compared with those treated with the pre-immune serum (Figure 2C). The inhibition rate was 6.9% [17.0 ± 2.3 and 15.8 ± 2.5 per 200× field for pre- and post-immune sera, respectively; *t*_(2)_ = 1.195, *P* = 0.355] at the 1:1000 dilution, 6.8% [16.6 ± 2.1 and 15.5 ± 2.8 per 200× field for pre- and post-immune sera, respectively; *t*_(2)_ = 2.046, *P* = 0.177] at the 1:500 dilution, and 6.7% [16.7 ± 1.7 and 15.5 ± 2.8 per 200× field for pre- and post-immune sera, respectively; *t*_(2)_ = 1.288, *P* = 0.327] at the 1:200 dilution. The mean parasite load for the control cultures receiving no serum treatment was 18.6 ± 1.1 per 200× field.

### Anti-cryptosporidial Effects of Candidate Compounds From Molecular Docking of *Cp*CDPK3

**FIGURE 3:**
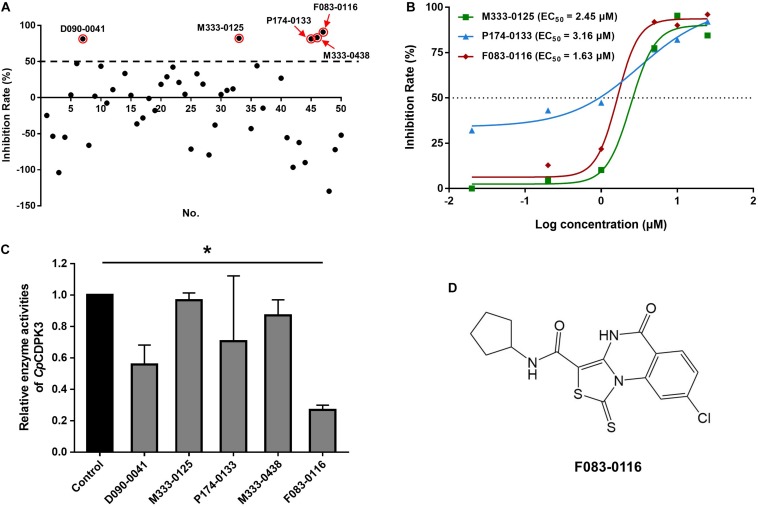
Inhibitory efficacy on *C. parvum* development *in vitro* by candidate compounds from *Cp*CDPK3 docking. **(A)** Efficacy of all 46 compounds at 10 μM obtained from primary screening (black dots). Five compounds with high levels of efficacy (>50%) were highlighted with red circles. Codes of the compounds are given in Table 1. Data presented are mean from three biological replicates. **(B)** Dose–response curves and EC_50_ values of anti-cryptosporidial efficacy by compounds M333-0125, P174-0133, and F083-0116. The response curves of D090-0041 and M333-0438 could not be drawn due to the rapid decay in efficiency at lower concentrations. Data presented are mean from three biological replicates. **(C)** Relative enzyme activities of *Cp*CDPK3 after treatment with five compounds. Data from the group treated with DMSO were used for data normalization. Only F083-0116 showed significant inhibitory effect on *Cp*CDPK3 (*P < 0.05). **(D)** Structure of F083-0116.

A total of 46 compounds were selected based on results of molecular docking of *Cp*CDPK3. They were evaluated for inhibition of *C. parvum* development (including both invasion and growth) at the concentration of 10 μM using a qPCR-based quantitation of parasite load in HCT-8 cell cultures. The mean inhibition rates of these compounds compared with the DMSO-treated controls ranged widely from -129.8 to 91.0% (Figure 3A). Using a cutoff value of 50%, only five compounds (M333-0125, P174-0133, F083-0116, D090-0041, and M333-0438) showed effects on *C. parvum* development (Figure 3A).

The efficacy of these five compounds was further assessed in dose-response experiments. All of them had inhibition rates greater than 80% at the concentration of 10 and 25 μM. Among them, the EC_50_ values were 2.45, 3.16, and 1.63 μM for M333-0125, P174-0133, and F083-0116, respectively (Figure 3B). Due to the rapid decay of the inhibitory effects of D090-0041 and M333-0438, their EC_50_ values could not be reliably calculated. The compound F083-0116 did not have any cytotoxicity on the growth of HCT-8 cells, with inhibition rates ranging from −0.62 to 12.82% at concentrations from 20 nM to 25 μM.

### Effects of Candidate Inhibitors on Enzyme Activity of *Cp*CDPK3

**FIGURE 4:**
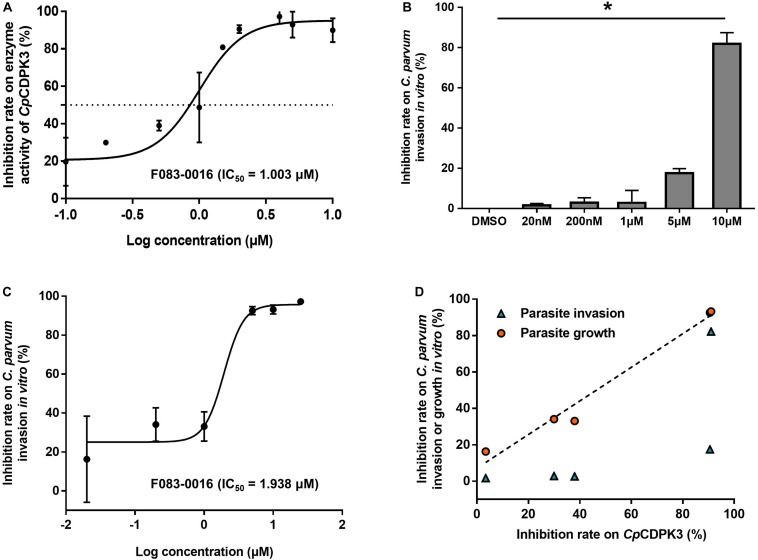
Inhibition of enzyme activity of *Cp*CDPK3 and the invasion and growth of *C. parvum*. **(A)** Dose–response curves and IC_50_ value of compound F083-0116 on *Cp*CDPK3 enzyme activity. Data presented are mean ± SD from three independent assays. **(B)** Neutralization efficiency of F083-0116 against *Cp*CDPK3 in *C. parvum* culture. Data from the group treated with DMSO were used for data normalization. **(C)** Dose–response curves of compound F083-0116 on *C. parvum* growth. Data presented are mean ± SD from three independent assays. **(D)** The correlation between inhibition rates of F083-0116 on *Cp*CDPK3 activity and *C. parvum* invasion or growth as measured using the Pearson *χ*^2^ goodness-of-fit test. *P < 0.05.

The enzymatic activity of *Cp*CDPK3 was measured with an NADH-coupled enzyme assay. At 30°C and pH 7.2, the catalytic efficiency of *Cp*CDPK3 was about 2368.2 nmol/mg/min, with the K_cat_ of 152.3 min^–1^. For the Syntide-2 used in the enzyme assay, the Michaelis constant (K_m_-_Syntide__–2_) of *Cp*CDPK3 was 76.8 ± 10.9 μM. When the five compounds were evaluated for their inhibitory effects on the enzyme activity of *Cp*CDPK3 *in vitro* at 5 μM, only F083-0116 had a significant effect (inhibition rate = 73.2%, *P* = 0.019, *t* = 33.518) (Figure 3C). The structure of compound F083-0116 is shown in Figure 3D. In dose–response evaluations of F083-0116, the IC_50_ was 1.00 μM (Figure 4A), with a rapid decay of inhibitory effect between 1 and 2 μM.

### Effects of Compound F083-0116 on *C. parvum* Invasion and Growth

In a further evaluation of the anti-cryptosporidial effect of F083-0116 using an invasion assay, the compound exhibited significant inhibition of *C. parvum* invasion only at 10 μM (inhibition rate = 82.2%, *P* = 0.023) (Figure 4B). Analysis of the data using the Pearson *χ*^2^ goodness-of-fit test had shown a poor correlation between the inhibition of enzyme activity and parasite invasion (*P* = 0.1742) (Figure 4D). In the evaluation of the anti-cryptosporidial effect of F083-0116 using a growth assay, the inhibitory effects of the compound on parasite load increased with the concentration of the compound used, with an EC_50_ of 1.94 μM (Figure 4C). The result of Pearson *χ*^2^ goodness-of-fit test of the data indicated that there was a significant and positive correlation between the inhibition of enzyme activity and parasite growth (*P* = 0.0016) (Figure 4D), with a Pearson’s correlation coefficient (*r*) of 0.9879.

## Discussion

Results of the study indicate that *Cp*CDPK is another CDPK candidate that could be potentially exploited for the development of effective treatments against cryptosporidiosis. Prior to this, studies on *Cp*CDPKs have focused exclusively on *Cp*CDPK1, which has a distinct structure compared with other CDPKs owing to the presence of a smaller gatekeeper, glycine (compared to methionine in others), in its ATP-binding pocket. In the present study, we have evaluated for the first time the function of *Cp*CDPK3. Our results suggest that *Cp*CDPK3 protein could play an essential role in the growth but not the invasion of *C. parvum*.

Several lines of evidence support the involvement of *Cp*CDPK3 in the growth of *C. parvum*. Our results suggest that *Cp*CDPK3 could have exerted its effect in the merozoites stage of *C. parvum*. The expression of *Cp*CDPK3 showed a stage-specific pattern with high expression in meronts and merozoites. At the RNA level, the expression of the *cgd5_820* gene peaked at 12 and 48 h post-infection, coinciding with the emergence of immature meront (12 h) and merozoite reinfection (48 h) period of *C. parvum* (Hijjawi et al., 2001; Mauzy et al., 2012). Previously RNA-Seq analysis revealed that most *C. parvum* genes, including CDPKs genes, are expressed in a stage-specific manner (Etzold et al., 2014; Lippuner et al., 2018). At the protein level, *Cp*CDPK3 appears to be present in the entire merozoites at 24 and 48 h post-infection. These results indicated that *Cp*CDPK3 might participate in the activities of merozoites, such as egress and gliding movements.

Results of the study indicate that *Cp*CDPK3 is probably not crucial in *C. parvum* invasion. In a *C. parvum* invasion assay, anti-*Cp*CDPK3 polyclonal antibodies failed to block the invasion of host by *C. parvum*. However, the neutralization assay with antibodies might not to be an optimal way to evaluate *Cp*CDPK3, as it has an intracellular localization. As an alternative, similar work with a candidate *Cp*CDPK3 inhibitor F083-0116 was performed. The compound produced partial inhibition of *C. parvum* invasion only at 10 μM, with poor correlation between the inhibition of enzyme activity and parasite invasion. In an assessment of the involvement of *Cp*CDPK3 in *C. parvum* growth, however, F083-0116 produced a significant correlation between the inhibitory effects on *Cp*CDPK3 enzyme activity and *C. parvum* growth. Together with the unique pattern of gene and protein expression, these findings suggest that *Cp*CDPK3 might be involved in *C. parvum* growth but not invasion.

The role of *Cp*CDPK3 in *C. parvum* growth appears to be similar to the function of *Pf*CDPK1 and *Tg*CDPK3, which clustered together with *Cp*CDPK3 in previous phylogenetic analyses of the kinase domains of apicomplexan CDPKs (Billker et al., 2009; Artz et al., 2011). *Pf*CPKD1 is reportedly an essential enzyme in the egress of *P. falciparum* merozoites, while *Tg*CDPK3 is known to regulate the egress of the parasite out of host cells (Mccoy et al., 2012; Kadian et al., 2017). Therefore, Green et al. identified two substrates of *Pf*CDPK1: myosin A tail domain-interacting protein (MTIP) and glideosome-associated protein 45 (GAP45), which are components of the motor complex involved in the invasion and egress of *P. falciparum* merozoites. Transcriptomic data indicate that *Pf*CDPK1 is mostly expressed in the meront stage (Le Roch et al., 2003). An inhibitor of *Pf*CDPK1, a 2,4,6-trisubstituted purine compound, inhibited merozoite egress from meronts in cell culture (Kato et al., 2008). Similarly, the inhibition of *Pf*CDPK1 by conditional expression of its auto-inhibitory J domain was reported to arrest parasite development late in the cell cycle during early merogony (Azevedo et al., 2013). The expression of *Pf*CDPK1 protein in *T. gondii* complemented the *Tg*CDPK3 mutant strain, rescuing its egress process (Gaji et al., 2014). In *T. gondii*, *Tg*CDPK3 is known to regulate its egress from host cells and under some conditions, microneme secretion, and motility, but not invasion (Lourido et al., 2012; Mccoy et al., 2012). *Tg*CDPK3 was further shown to be an upstream regulator of other calcium-dependent signaling pathways, suggesting that the function of *Tg*CDPK3 is not limited to regulating egress (Treeck et al., 2014). The sequence similarity to *Pf*CDPK1 and *Tg*CDPK3 by *Cp*CDPK3 suggests that it might play a similar role in the egress of *C. parvum* merozoites.

Through *Cp*CDPK3 is most related to *Cp*CDPK1 among CDPKs from *C. parvum*, the functions of these two enzymes are probably different. Thus, both *Cp*CDPK1 and *Cp*CDPK3 have similar structures with one kinase domain and four EF-hands and therefore share the same activation mechanisms (Billker et al., 2009; Wernimont et al., 2011). However, *Cp*CDPK1 has been shown to be involved in *C. parvum* invasion rather than growth (Castellanos-Gonzalez et al., 2016; Kuhlenschmidt et al., 2016). Although a recent study has shown that some candidate *Cp*CDPK1 inhibitors could potently inhibit *C. parvum* growth *in vitro*, there was no apparent correlation between anti-*Cp*CDPK1 activities and *C. parvum* growth inhibition (Huang et al., 2017). In contrast, data generated with candidate inhibitors in the present study suggest that *Cp*CDPK3 is involved in *C. parvum* growth but not invasion.

Thus far, the design of inhibitors for *Cp*CDPKs has focused on pyrazolopyrimidine (PP) analogs and bumped kinase inhibitors (BKIs), which target *Cp*CDPK1. The unique smaller gatekeeper in the ATP-binding pocket makes *Cp*CDPK1 sensitive to PP analogs and BKIs. In contrast, *Cp*CDPKs do not have the gatekeeper, thus are not susceptible to these compounds (Wernimont et al., 2010; Artz et al., 2011; Zhang et al., 2014). As a result, studies on *Cp*CDPK3 inhibitors are extremely limited. In this study, five compounds from the molecule docking of *Cp*CDPK3 were shown to inhibit *C. parvum* development *in vitro*, but only F083-0116 (8-chloro-*N*-cyclopentyl-5-oxo-1-thioxo-4,5-dihydro-1*H*-thiazolo[3,4-*a*]quinazoline-3-arboxamide) had the ability to inhibit the enzyme activity of *Cp*CDPK3. Although M333-0125 and P174-0133 had similar efficiency in inhibiting parasite development, they failed to inhibit the enzyme activity of *Cp*CDPK3, indicating that these two compounds might exert their anti-cryptosporidial effects through other mechanisms.

## Conclusion

Our findings suggest that *Cp*CDPK3 plays an essential role in the growth of *C. parvum*, and inhibitors of the enzyme can be potential candidates for the treatment of cryptosporidiosis. They need to be supported by additional studies using more advanced tools such as gene complementation, ablation, and conditional knockdown. Additional screening of *Cp*CDPK3 inhibitors and better understanding of their action mechanisms are also needed to make *Cp*CDPK3 an ideal target in the development of new drugs against cryptosporidiosis.

## Data Availability Statement

All datasets generated for this study are included in the article/supplementary material.

## Author Contributions

YF and LX designed the study. QZ, JS, and RX performed the experiments and statistical analysis. YL and ZZ performed the molecular docking work. NL and YG provided technical assistance. QZ, YF, and LX developed the manuscript. All authors approved the final version for publication.

## Conflict of Interest

The authors declare that the research was conducted in the absence of any commercial or financial relationships that could be construed as a potential conflict of interest.

## Footnotes

1 https://imagej.nih.gov/ij/

2 https://www.graphpad.com
